# Proteomic and Metabolomic Correlates of Healthy Dietary Patterns: The Framingham Heart Study

**DOI:** 10.3390/nu12051476

**Published:** 2020-05-19

**Authors:** Maura E. Walker, Rebecca J. Song, Xiang Xu, Robert E. Gerszten, Debby Ngo, Clary B. Clish, Laura Corlin, Jiantao Ma, Vanessa Xanthakis, Paul F. Jacques, Ramachandran S. Vasan

**Affiliations:** 1 Section of Preventive Medicine and Epidemiology, Department of Medicine, Boston University School of Medicine, Boston, MA 02118, USA; laura.corlin@tufts.edu (L.C.); vanessax@bu.edu (V.X.); vasan@bu.edu (R.S.V.); 2 Department of Epidemiology, Boston University School of Public Health, Boston, MA 02118, USA; rsong@bu.edu; 3 Department of Mathematics and Statistics, Boston University College of Arts and Sciences, Boston, MA 02215, USA; xiangx@bu.edu; 4 Division of Cardiovascular Medicine Beth Israel Deaconess Medical Center, Boston, MA 02215, USA; rgerszte@bidmc.harvard.edu (R.E.G.); dngo@bidmc.harvard.edu (D.N.); 5 Broad Institute of Massachusetts Institute of Technology and Harvard, Cambridge, MA 02142, USA; clary@broadinstitute.org; 6 Department of Public Health and Community Medicine, Tufts University School of Medicine, Boston, MA 02111, USA; 7 Framingham Heart Study, Framingham, MA 01702, USA; jiantao.ma@tufts.edu; 8 Nutrition Epidemiology and Data Science, Tufts University Friedman School of Nutrition Science and Policy, Boston, MA 02111, USA; paul.jacques@tufts.edu; 9 Department of Biostatistics, Boston University School of Public Health, Boston, MA 02118, USA; 10 Nutrition Epidemiology, Jean Mayer USDA Human Nutrition Research Center on Aging at Tufts University, Boston, MA 02111, USA; 11 Section of Cardiovascular Medicine, Department of Medicine, Boston University School of Medicine, Boston, MA 02118, USA; 12 Center for Computing and Data Sciences, Boston University, Boston, MA 02215, USA

**Keywords:** dietary patterns, diet quality, proteomic, metabolomic, biomarker

## Abstract

Data on proteomic and metabolomic signatures of healthy dietary patterns are limited. We evaluated the cross-sectional association of serum proteomic and metabolomic markers with three dietary patterns: the Alternative Healthy Eating Index (AHEI), the Dietary Approaches to Stop Hypertension (DASH) diet; and a Mediterranean-style (MDS) diet. We examined participants from the Framingham Offspring Study (mean age; 55 years; 52% women) who had complete proteomic (*n* = 1713) and metabolomic (*n* = 2284) data; using food frequency questionnaires to derive dietary pattern indices. Proteins and metabolites were quantified using the SomaScan platform and liquid chromatography/tandem mass spectrometry; respectively. We used multivariable-adjusted linear regression models to relate each dietary pattern index (independent variables) to each proteomic and metabolomic marker (dependent variables). Of the 1373 proteins; 103 were associated with at least one dietary pattern (48 with AHEI; 83 with DASH; and 8 with MDS; all false discovery rate [FDR] ≤ 0.05). We identified unique associations between dietary patterns and proteins (17 with AHEI; 52 with DASH; and 3 with MDS; all FDR ≤ 0.05). Significant proteins enriched biological pathways involved in cellular metabolism/proliferation and immune response/inflammation. Of the 216 metabolites; 65 were associated with at least one dietary pattern (38 with AHEI; 43 with DASH; and 50 with MDS; all FDR ≤ 0.05). All three dietary patterns were associated with a common signature of 24 metabolites (63% lipids). Proteins and metabolites associated with dietary patterns may help characterize intermediate phenotypes that provide insights into the molecular mechanisms mediating diet-related disease. Our findings warrant replication in independent populations

## 1. Introduction

Sub-optimal diet quality is a leading cause of death in the United States and is estimated to contribute to approximately 44% of coronary heart disease deaths and 51% of stroke related deaths [1]. Data from clinical trials and prospective cohort studies indicate that healthy dietary patterns are associated with better metabolic health, lower risk of major chronic diseases, and lower mortality [2,3,4,5]. These studies indicate that diet quality may be a key factor in the prevention and mitigation of chronic disease. Yet, the molecular mechanisms [2,3] underlying the beneficial effects of healthy diet are not completely understood.

The identification of molecular biomarkers (proteins and metabolites) related to dietary patterns holds promise to elucidate biological pathways underlying the diet-related risk of chronic disease. Molecular biomarkers related to dietary intake may also relate to risk factors for chronic disease, or overt chronic disease per se. Such molecular markers reflecting dietary patterns have the potential to help inform risk assessment and allow for targeted preventive measures prior to the onset of chronic disease states. Population-based high-throughput proteomic studies of dietary patterns are lacking even though proteomic profiling may identify molecular biomarkers reflective of the biological functions/dysfunctions associated with an exposure (such as diet) or chronic disease states. In contrast, the use of untargeted metabolomics in nutrition research has increased in past years with the primary goal of biomarker discovery for the objective assessment of dietary intake [6]. Fewer studies have sought to relate dietary metabolomic profiles to disease outcomes; some studies have suggested that certain circulating metabolites may contribute to the biological underpinnings of diet–disease relations [7,8,9].

Multi-level omics analyses that combine high-dimensional molecular data from high throughput platforms can provide a comprehensive assessment of intermediate phenotypes (e.g., molecular endophenotypes) that may help link diet (or other exposures) to more distal chronic disease phenotypes. We hypothesized that proteomic and metabolic signatures of habitual dietary patterns encompass the respective functional states and metabolic consequences of unique dietary patterns including the sequelae of chronic disease. The objective of the present investigation was to determine the associations of the Alternative Healthy Eating Index (AHEI), Dietary Approaches to Stop Hypertension (DASH) diet, and a Mediterranean-style diet (MDS) with 1373 plasma proteins and 216 circulating metabolites in a sample of community-dwelling middle-aged adults.

## 2. Materials and Methods

### 2.1. Study Sample

The description of the Framingham Offspring Study is located elsewhere [10]. For the present investigation, we evaluated data from participants who attended the fifth examination cycle (1991–1995) of the Framingham Offspring Study. Details of participant inclusion are displayed in Supplementary Figure S1. For our analyses of diet–protein relations, 1913 participants who had proteomic profiling completed on the SOMAscan platform were eligible. Participants were excluded from analysis if they did not have complete dietary data (*n* = 200) or had missing covariate data (*n* = 51). This resulted in a final analytical sample of 1662. For analyses of diet–metabolite relations, 2526 participants who had metabolites assayed were eligible. Participants were excluded from analysis if they did not have complete dietary data (*n* = 242) or if they were missing covariate data (*n* = 76). This resulted in a second analytical sample of 2208 for diet–metabolite relations.

### 2.2. Protein Quantification

Proteomics profiling in the Framingham Offspring Study has been described previously [11,12]. Blood samples were collected from participants at the fifth exam Heart Study visit using standard phlebotomy procedures. Using the SOMAscan platform, a total of 1373 proteins were quantified using single-stranded DNA-based aptamers. Samples were assayed in two batches (*n* = 821 and *n* = 1092). For each respective batch, age and sex adjusted protein values were log_e_ transformed and standardized to a mean = 0 and standard deviation (SD) = 1. The inter- and intra-assay reproducibility of proteins quantified on the SOMAscan platform in the Framingham Offspring Study has previously been reported [11].

### 2.3. Metabolite Quantification

Measurements of 216 metabolites in the Framingham Offspring Study has been previously described in detail [13,14,15]. Blood samples were collected at the fifth exam Heart Study visit. Positively charged polar, negatively charged polar, and lipid metabolites were quantified using liquid chromatography with tandem mass spectrometry (LC/MS/MS). Known standards were used to identify metabolites and internal standards were used for quality control [13]. Nomenclature for lipid metabolites includes the total lipid acyl chain-length followed by the total number of double bonds (e.g., twenty-two carbon acyl chain and six double bonds is indicated by = C22:6).

### 2.4. Dietary Assessment

Dietary intake at examination cycle five was assessed using the Harvard semi-quantitative food frequency questionnaire (FFQ). The Harvard FFQ measures usual frequency of consumption of 126 dietary items over the year preceding the Heart Study visit. Food frequency categories range from none or <1 serving per month to ≥ 6 servings per day. Use of the Harvard FFQ has previously been validated for the assessment of dietary intake using 7-day dietary records [16]. We only used FFQs that were considered valid (<13 blank items and estimated daily caloric intake was ≥600 kcal/d and <4000 kcal/d for women or <4200 kcal/d for men) [17].

### 2.5. Dietary Pattern Indices

The AHEI, DASH diet score, and MDS were constructed using dietary intake data from the aforementioned FFQ. Components and scoring criteria for each score were based on prior studies and have been described in detail elsewhere [18,19,20].

AHEI components include vegetables, fruits, nuts and legumes, sugar-sweetened beverages and fruit juice, whole grains, red and processed meat, eicosapentaenoic acid and docosahexaenoic acid, polyunsaturated fatty acids (PUFA), *trans* fatty acids, sodium, and alcohol [20]. For each component, a maximum score of 10 points was possible. Reverse scores (lower consumption receives a higher score) were assigned to sugar-sweetened beverages, *trans* fatty acids, and sodium. Individual components were summed to a maximum total score of 110 points.

The DASH diet score has 8 components, which includes fruits and fruit juices, vegetables, nuts and legumes, whole grains, low-fat dairy, sodium, red and processed meats, and sugar-sweetened beverages. For each component, scoring was based on quintiles of intake with the first quintile (Q1) receiving a score of 1 and the fifth quintile (Q5) receiving the maximum score of 5 points [19]. Reverse scores were assigned to sodium, red and processed meats, and sugar-sweetened beverages. All components were summed to a maximum DASH diet score of 40.

Lastly, the MDS components included vegetables, fruits, nuts, legumes, whole grains, fish, red meat, ratio of monounsaturated fatty acids (MUFA) to saturated fatty acids (SFA), and alcohol. Except for alcohol, all component scores are based on sex-specific quartiles of intake for our respective sample, with participants in the first quartile (Q1) receiving a score of 0 and the fourth quartile (Q4) having the maximum score of 3 [21]. Reverse scores were assigned to red and processed meat component. Participants received a score of 1 for sex-specific moderate alcohol consumption or a score of 0 for over- or under-consumption [18]. Component scores were summed to a maximum final MDS score of 25.

### 2.6. Covariate Assessment

We included the following covariates in our analysis: age, sex, total caloric intake, current smoking status, physical activity index, use of lipid lowering medication, use of anti-hypertensive medication, and body mass index (BMI). All covariates were assessed at the fifth examination cycle of the Framingham Offspring Study (1991–1995). We classified participants who smoked regularly in the year preceding the Heart Study visit as current smokers. Use of anti-hypertensive and lipid-lowering medications in the past year was based on self-report. BMI was calculated as measured weight in kilograms divided by the square of height in meters (kg/m^2^). The physical activity index was calculated based on time and intensity of activities in a day [22]. Lastly, total energy was calculated from the aforementioned semi-quantitative FFQ.

### 2.7. Statistical Analysis

We used multivariable linear regression models to determine the cross-sectional associations of each dietary pattern index (AHEI, DASH, and MDS; independent variables, a separate model for each) with each protein and metabolite (dependent variables, a separate model for each). We standardized all three dietary pattern indices (mean = 0, SD = 1) and modeled the standardized scores as continuous variable to maximize our statistical power. Results are reported as increments in proteins or metabolite concentrations for each SD-unit increase in the standardized dietary pattern indices (AHEI, DASH or MDS). Multivariable linear regression models included adjustment for age, sex, total caloric intake, current smoking status, physical activity index, use of lipid lowering medication, use of anti-hypertensive medication, and BMI. We calculated age- and sex-adjusted Spearman’s correlation coefficients among proteins and metabolites (separately) identified as statistically significant in our second multivariable regression model.

For all analyses we considered the Benjamini–Hochberg false discover rate (FDR) q value of ≤0.05 to define statistical significance. A Bonferroni adjustment (0.05/1373 proteins and 0.05/216 metabolites) was used to display a condensed list of top significant proteins and metabolites. Statistical analyses were completed using SAS statistical software (version 9.4; SAS Institute, Cary, NC, USA) and R (version 3.6.1) run on RStudio (RStudio: Integrated Development for R. RStudio, Inc., Boston, MA, USA).

### 2.8. Enrichment Analysis

We completed an enrichment analysis to determine underlying biological significance of proteins that have statistically significant associations with dietary patterns. A pathway over-representation analysis was conducted on significant (FDR q ≤ 0.05) proteins using Web-based Gene Set Analysis Toolkit (WebGestalt) [23]. Proteins were mapped to the Kyoto Encyclopedia of Genes and Genomes (KEGG) functional database [24]. KEGG pathways with less than 5 proteins or more than 2000 proteins were excluded from the analysis. Enriched pathways with an FDR q ≤ 0.05 were considered statistically significant. Significant metabolites were mapped to the KEGG functional database for descriptive annotation.

#### Overlap between Dietary Protein and Metabolite Quantitative Trait Loci and Prior Genome-Wide Association Study Risk Loci 

Previous work in the Framingham Offspring Study has identified genetic loci (protein and metabolite quantitative trait loci [p/mQTL]) of 156 of the proteins and all of the 216 metabolites assayed at examination cycle five [25,26]. Genotyping and the identification of p/mQTLs in the Framingham Offspring Study have been described elsewhere [25,26]. In the present study, we present p/mQTL that were previously identified in the Framingham Offspring Study to be associated with proteins and/or metabolites that we observed to be significantly associated with at least one of the respective dietary patterns. We searched the NHGRI-EBI Catalogue of Published genome-wide association studies (GWAS) [27] to determine overlap between dietary p/mQTL and risk loci identified in prior GWAS.

## 3. Results

### 3.1. Sample Characteristics

Our overall study sample had an average age of 55 years and 52 percent were women. Sex-specific participant characteristics, represented as mean (SD) or frequency (proportion), are displayed in Table 1. On average, women had a lower BMI and a small proportion reported use of lipid-lowering and anti-hypertensive medications, compared to men. Additionally, women reported a lower average energy intake and had higher average AHEI and DASH dietary pattern scores.

### 3.2. Associations of Dietary Patterns with Plasma Protein Concentrations

**Figure 1:**
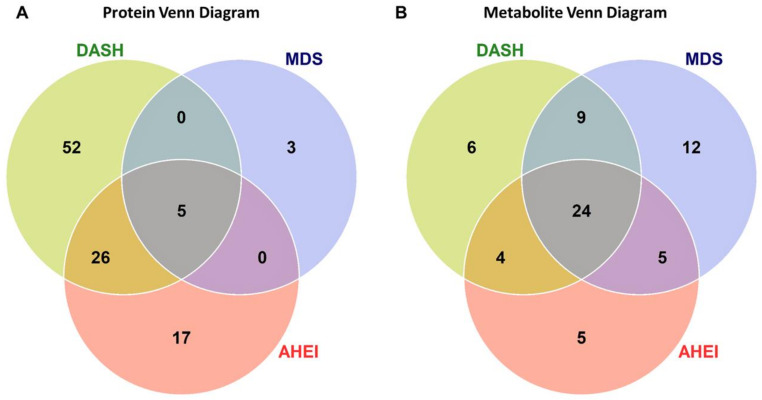
The number of protein (**A**) and metabolite (**B**) markers associated with dietary pattern indices. Venn diagrams illustrate the overlap of significant proteins (FDR q ≤ 0.05) and metabolites across the AHEI, DASH, and MDS dietary patterns. Significant proteins and metabolites were based on a false discovery rate threshold ≤ 0.05 from multivariable models adjusted for age, sex, total caloric intake, current smoking, physical activity index, lipid lowering medication, anti-hypertensive medication, and body mass index. Abbreviations: AHEI, Alternative Healthy Eating Index; DASH, Dietary Approaches to Stop Hypertension; FDR, false discovery rate; MDS, Mediterranean-style Diet Score.

**Figure 2:**
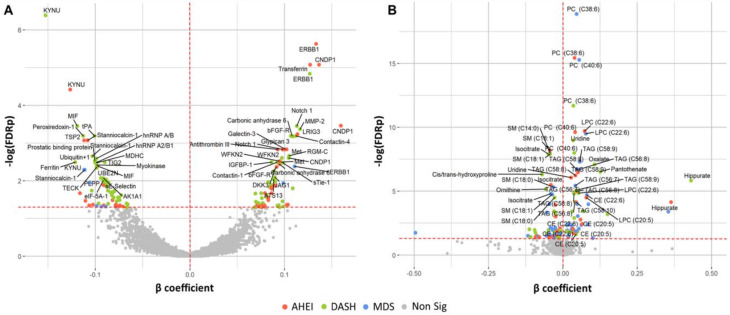
Protein (**A**) and metabolite (**B**) markers associated with dietary pattern indices. Colored points (red, green, blue) indicate statistical significance (FDR q ≤ 0.05) by the respective dietary pattern indices. Multivariable regression models are adjusted for age, sex, total caloric intake, current smoking, physical activity index, lipid lowering medication, anti-hypertensive medication, and body mass index. β estimates represent the change in marker per one-unit increase in the respective dietary pattern indices. Eleven metabolites with β coefficients < −0.50 or >0.50 are excluded from the figure. Abbreviations: AHEI, Alternative Healthy Eating Index; CE, cholesterol ester; DASH, Dietary Approaches to Stop Hypertension; FDR, false discovery rate; LPC, lysophosphatidylcholine; LPE, lysophosphatidylethanolamine; MDS, Mediterranean-style Diet Score. PC, phosphatidylcholine; SM, sphingomyelin; TAG, triacylglycerol.

**Figure 3:**
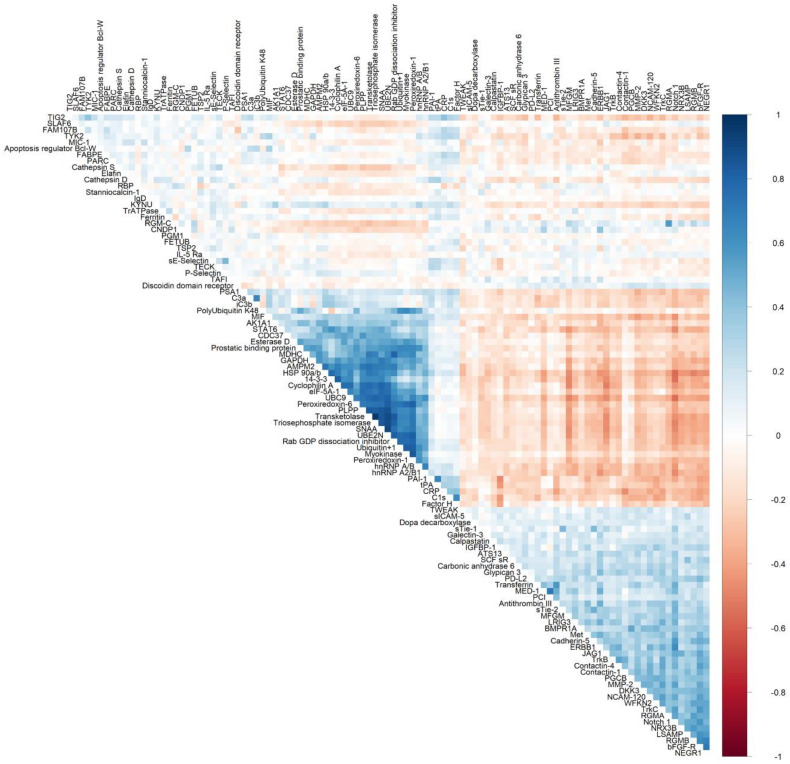
Heatmap depicting Spearman’s partial correlation coefficients adjusted for age and sex between proteins that were statistically significant (FDR p ≤ 0.05) in multivariable models adjusting for age, sex, total caloric intake, current smoking, physical activity index, lipid lowering medication, anti-hypertensive medication, and body mass index.

Overall, 103 unique proteins were significantly associated with at least one dietary pattern index (48 with AHEI, 83 with DASH, and 8 with MDS; Figure 1A). Forty-six proteins were directly associated with at least one dietary pattern index and 57 proteins had inverse associations with at least one dietary pattern index (Figure 2A). The top significant results by a Bonferroni adjustment are listed in Table 2. Beta coefficients representing the difference in concentrations of each of the 103 proteins per SD increase in the AHEI, DASH and MDS indices are listed in Supplemental Table S1. Five proteins (epidermal growth factor receptor [ERBB1], kynureninase [KYNU], stanniocalcin 1, macrophage migration inhibitory factor [MIF], and WAP, kazal, immunoglobulin, kunitz and NTR domain-containing protein 2 [WFKN2]) were significantly associated with all three dietary pattern indices. We observed that 52 proteins were uniquely associated with the DASH diet score (19 direct and 33 inverse associations; all FDR q ≤ 0.05) and 17 proteins were uniquely associated with the AHEI (7 direct and 10 inverse associations; all FDR q ≤ 0.05). Additionally, 26 proteins were significantly associated with the DASH diet score and the AHEI but not the MDS (15 direct and 11 inverse associations; all FDR q ≤ 0.05). Unlike the DASH diet score and the AHEI, associations of the MDS with plasma proteins were less evident. The MDS was significantly associated with just 8 proteins, 3 of which (p-selectin, intercellular adhesion molecular 5 [ICAM-5], and cathepsin S) uniquely related to the MDS (all FDR q ≤ 0.05). Among the 103 unique proteins, we observed two groupings of positively correlated proteins (34 proteins and 40 proteins; Figure 3). The first grouping consists of 34 proteins that primarily had inverse associations with dietary patterns and the second grouping consists of 40 proteins that primarily had positive associations with dietary patterns.

### 3.3. Associations of Dietary Patterns with Plasma Metabolite Concentrations

**Figure 4:**
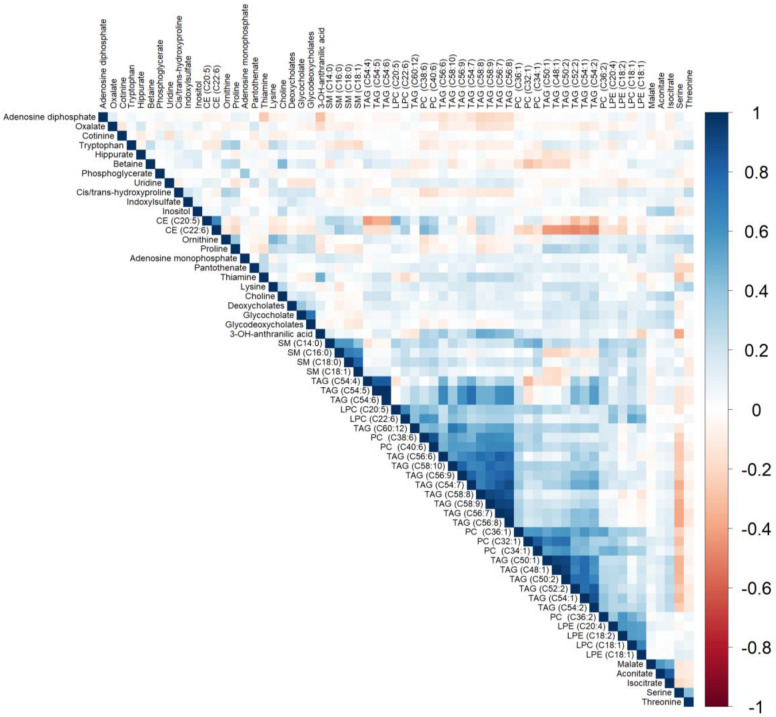
Heatmap depicting Spearman’s partial correlation coefficients adjusted for age and sex between metabolites that were statistically significant (FDR *p* ≤ 0.05) in multivariable models adjusting for age, sex, total caloric intake, current smoking, physical activity index, lipid lowering medication, anti-hypertensive medication, and body mass index. CE, cholesterol ester; FDR, false discovery rate; LPC, lysophosphatidylcholine; LPE, lysophosphatidylethanolamine; PC, phosphatidylcholine; SM, sphingomyelin; TAG, triacylglycerol.

Of the 216 plasma metabolites, 65 were associated with at least one dietary pattern index (38 with AHEI, 43 with DASH, 50 with MDS; all FDR q ≤ 0.05; Figure 1B and Figure 2B). Of these, 27 metabolites had direct associations and 38 metabolites had inverse associations with a dietary pattern. The top statistically significant results after a Bonferroni correction are listed in Table 3. Beta coefficients representing the difference in concentrations of each of the 65 proteins per one-unit increase in the AHEI, DASH and MDS indices are listed in Supplemental Table S2. The majority of statistically significant metabolites associated with the three dietary patterns were lipids (55%). Additional notable classes of molecules associated with the three dietary patterns included amino acids (11%), bile acids and derivatives (5%), nucleotide metabolism (5%), and tricarboxylic acid and derivatives (5%). We observed that 24 metabolites were significantly associated (15 directly and 9 inversely) with all three dietary pattern indices (all FDR q ≤ 0.05). Half of the 24 metabolites were highly-unsaturated lipid species directly associated with the respective diet indices. Overall, we observed fewer significant unique associations of the three dietary pattern indices with metabolites than with proteins. The MDS was uniquely associated with 11 lipid species and the nucleotide metabolite adenosine monophosphate (AMP, all FDR q ≤ 0.05). The AHEI and DASH indices had fewer unique associations (5 and 6 respectively), all of which were with non-lipid metabolites (all FDR q ≤ 0.05). Of the 65 metabolites, we observed that concentrations of lipid species with a similar degree of saturation had direct correlations with each other (Figure 4).

### 3.4. Protein Enrichment Analysis

We completed a pathway over-representation analysis of the 103 proteins significantly associated with the AHEI, DASH, or MDS diet indices. Sixty-eight proteins successfully mapped to KEGG pathways. The top ten enriched pathways for these 68 proteins are displayed in Table 4. Seven pathways were significantly enriched by protein related to dietary patterns: complement and coagulation cascades, staphylococcus aureus infection, endocrine resistance, HIF-1 signaling pathway, central carbon metabolism in cancer, PI3K-Akt signaling pathway, and malaria (all FDR q ≤ 0.05). Additionally, three pathways had FDR q < 0.07, including fluid shear stress and atherosclerosis, Prostate cancer, and cell adhesion molecules (CAMs). Pathways were broadly involved in biological processes such as cellular proliferation, cellular metabolism, immune response, and inflammation. We completed separate analyses on the 48 proteins significantly associated with the AHEI and the 83 proteins significantly associated with the DASH diet score (Supplementary Table S3). Thirty-seven of the 48 proteins associated with the AHEI and 49 of the 83 protein associated with the DASH diet score successfully mapped to KEGG pathways. This secondary enrichment analysis suggested that proteins associated with the DASH diet score predominately enriched the HIF-1 signaling pathway, PI3K-Akt signaling pathway, malaria, fluid shear stress and atherosclerosis, and prostate cancer pathways.

### 3.5. Metabolite Enrichment

In a descriptive assessment, we mapped metabolites to pathways in the KEGG functional database (Supplemental Table S4). Metabolites mapped to notable pathways including bile secretion, cholesterol metabolism, and ABC transporters. Additional pathways include choline metabolism, glycerophospholipid metabolism, and vitamin digestion and absorption.

### 3.6. Overlap between Dietary Protein and Metabolite Quantitative Trait Loci and Prior Genome-Wide Association Study Risk Loci

Of the previously identified genetic loci associated with 156 proteins from the SOMAscan platform, 39 were related with concentrations of 25 plasma proteins that we identified as having a significant association with at least one respective dietary pattern index (Table 5). Four of these dietary pQTL were associated with clinical traits in prior GWAS analyses. In particular, a variant (rs2519093) of the *ABO* gene, which is directly associated with concentrations of E-selectin, has direct associations with coronary artery disease [28], venous thromboembolism [29], and cardiometabolic risk factors. Additionally, 19 mQTL were associated with concentrations of 18 metabolites, 15 of which are lipid metabolites (Table 6). Many lipid metabolites were associated with genetic variants of the of the fatty acid desaturase gene family [*FADS1-2*]. Genetic variants of the FADS gene cluster are associated with plasma concentrations of omega-3 and -6 fatty acids [30,31], and cardiometabolic risk factors (blood lipids, insulin, and glycemic markers) [32,33]. Further, a genetic variant of the *APOA* gene cluster was associated with concentrations of TAG (C52:4) and has been linked to increased susceptibility of CVD [34].

## 4. Discussion

Proteomic and metabolomic correlates of dietary patterns (sometimes referred to as ‘signatures’) may collectively represent an intermediate phenotype of the underlying metabolic state and provide insight into the molecular mechanisms mediating diet and chronic disease associations. In the present investigation we identified high-throughput proteomic *and* metabolomic signatures of dietary patterns that have been previously associated with reduced risk of chronic disease mortality from all causes [4,5]. Our main findings are four-fold; first, we identified unique proteomic correlates of the DASH and AHEI dietary patterns. Second, we identified a shared ‘signature’ of 24 metabolites associated with all three healthy dietary patterns. Third, downstream enrichment analysis indicated that proteins associated with dietary patterns are involved in biological pathways that may underlie associations between dietary patterns and metabolic health. Fourth, overlap between dietary p/mQTL and prior GWAS risk loci suggest the potential utility of dietary pattern-related proteins and metabolites as endophenotypes that may link diet to cardiometabolic health and risk of chronic disease.

Our investigation adds to the literature by relating a broad array of over 1300 plasma proteins to dietary patterns using multiplexed high-throughput single-stranded DNA aptamer technology that allows for efficient and deep profiling of the plasma proteome [35]. Of the 103 dietary pattern-related proteins in our investigation, 16 were associated with various metabolic traits (BMI, visceral adiposity, triglycerides, insulin resistance, and fasting glucose) in the Diet, Obesity, and Genes interventional trial [36,37] and 45 proteins were associated with cardiometabolic risk and/or incidence CVD in the Framingham Offspring Study [11]. In addition, our over representation analysis indicated that diet related proteins enriched biological pathways involved in cellular proliferation/metabolism and immune response/inflammation. However, we observed considerable overlap of proteins among the identified pathways. The relatively modest number of proteins that successfully mapped to KEGG pathways may have hindered a thorough enrichment analysis. Lastly, our observation that concentrations of E selectin were related to a poorer DASH diet score and a variant of the *ABO* gene that is directly associated with CAD may delineate a functional link between diet and metabolic health.

To the best of our knowledge, only two prior studies have examined proteomic profiles of dietary patterns [38,39]. A recent investigation by Warensjo Lemming et al. examined a panel of 184 proteins and identified 59 proteins associated with Swedish population empirically derived dietary patterns; 21 of which were validated in an independent cohort [39]. We observed significant positive associations with five of the 21 proteins (ERBB1 [EGFR], insulin like growth factor binding protein 1 [IGFBP1], programmed death ligand 2 [PDL2], galectin-3 [Gal3], and contactin 1 [CNTN1]) that are involved in processes including cellular metabolism, cellular proliferation, immune response, and cellular adhesion. Prior experimental studies indicate that gene and/or protein expression of ERBB1, PDL2, and CNTN1 is atheroprotective [40,41,42]. Further, multiple studies have reported that low concentrations of IGEBP1 are directly associated with glucose intolerance and risk of diabetes mellitus [43,44]. Notably, ERBB1 and CNTN1 were found to be inversely associated with cardiometabolic risk and/or incidence CVD [11,45]. In contrast, prior work has identified direct associations between Gal3 and heart failure [46]. Thus, the direct association of Gal3 with healthy dietary patterns warrants further investigation.

Perhaps our most intriguing finding is the unique proteomic signatures, or lack thereof, we observed across dietary patterns. Notably, the DASH diet score was associated with a unique signature of 52 proteins, which enriched pathways involved in cellular metabolism, hypoxia, inflammation, and atherosclerosis. Clinical trials have demonstrated the efficacy of the DASH diet in lowering blood pressure [3] and epidemiological studies have found that adherence to the DASH diet is associated with reduced risk of hypertension, CVD, and mortality [19,47,48]. Additional studies are needed to determine if proteins in this unique signature are associated with blood pressure lowering and other cardio-protective qualities of the DASH diet.

Similar to our investigation, two prior studies comparing hypothesis-driven dietary patterns reported just a modest number of unique dietary pattern and metabolite associations [49,50]. A higher degree of similarities across metabolomic signatures of healthy dietary patterns might indicate a generalized metabolic response of an overall healthy dietary pattern. Prior studies have reported that a large proportion of the metabolites associated with dietary patterns are lipids [49,50,51]. Lipids represented 63% of the metabolites associated with all three dietary patterns. We observed that lipids directly associated with a higher diet quality tended to have ≥5 carbon double bonds. Further, we observed that genetic variants (rs174548 and rs174550) associated with distinct highly-unsaturated lipid species had concordant associations with blood triglycerides and high-density lipoprotein metabolism [52,53]. Previous studies have found that highly-unsaturated lipids, with a longer acyl-chain length have inverse associations with risk of CVD and diabetes mellitus [54,55,56,57]. Hence, lipid correlates of dietary patterns, particularly highly-unsaturated lipids, may serve as markers of overall diet quality and may be directly related to pathogenesis of cardiometabolic and other chronic disease risks.

Lastly, we note that concentrations of several metabolites associated with the respective dietary patterns could be influenced by composition of the gut microbiome. This includes metabolites involved in choline metabolism (phosphotidylcholine, choline, and betaine), bile acid metabolism (glycocholate, deoxycholate, and deoxyglycholoate), and amino acid metabolism (tryptophan, indoxylsufate, proline, and hydroxyproline). Such metabolites have previously been linked to inflammation, chronic kidney disease, and CVD [58,59,60,61]. Metabolites related to dietary patterns and the gut microbiome may represent the functional response of diet—microbial interactions that influence metabolic health, a premise that warrants further evaluation.

The present study has several strengths. The Framingham Offspring Study is a well-established cohort with a comprehensive assessment of phenotypic, lifestyle, and dietary data on participants. We used a multiplexed aptamer-based high-throughput assay to identify and quantify a large number of plasma proteins. Lastly, we assessed three complementary dietary pattern indices that are extensively reviewed in the literature and can be readily reproduced in other study populations. Limitations of our investigation include the use of self-reported dietary data, which is prone to measurement error that may lead to misclassification and attenuation of observed associations. A more comprehensive metabolomic profile would aid in direct comparisons across studies. A large proportion of the metabolites associated with dietary patterns were lipid species that do not map to the KEGG functional database. This precluded a thorough enrichment analysis of metabolites related to dietary patterns. Participants from the Framingham Offspring Study are predominantly white and of European ancestry. Hence, our results may not be generalizable across populations that are more racially/ethnically diverse. We conducted a cross-sectional analysis and, therefore, cannot determine causality of any of the observed associations, and cannot exclude the possibility of uncontrolled or residual confounding.

In summary, the present investigation identified proteomic and metabolomic correlates of three dietary patterns. The DASH and AHEI were associated with unique proteomic signatures; whereas the largest metabolomic ‘signature’ was associated with all three dietary patterns. Proteins and metabolites related to dietary patterns may have prognostic value and inform future clinical interventions. Additional work, in in vitro and in vivo settings, is warranted to identify genetic determinants of dietary correlates and examine how they may relate to the progression of modifiable chronic disease. Findings from this investigation will require replication in independent and ethnically diverse populations.

## Figures and Tables

**Table 1:** Sample characteristics at examination five of the Framingham Offspring Study.^1^

	Men	Women
*n* = 2208	Mean ± SD	Mean ± SD
	*n* = 1054	*n* = 1154
Age, yr	55.7 ± 9.9	54.5 ± 9.6
Current smoker, n (%)	190 (18%)	210 (18%)
Physical activity score	36.0 ± 7.4	33.4 ± 4.7
BMI, kg/m^2^	28.2 ± 4.1	26.7 ± 5.4
Lipid-lowering medications, n (%)	101 (10)	67 (6)
Anti-hypertensive medication, n (%)	238 (23)	195 (17)
Energy intake, kcal/day	1992 ± 642	1742 ± 566
AHEI (0–110)	48.0 ± 9.6	51.7 ± 9.7
DASH (0–40)	23.0 ± 4.9	25.3 ± 4.7
MDS (0–25)	12.3 ± 4.3	12.0 ± 4.4
Food Group Intake		
Vegetables, svg/d	2.0 ± 1.3	2.4 ± 1.7
Fruits, svg/d	1.2 ± 1.0	1.4 ± 1.2
Low-fat dairy, svg/d	0.8 ± 0.9	0.9 ± 1.0
Nuts, svg/wk	0.4 ± 0.6	0.2 ± 0.4
Legumes, svg/wk	0.3 ± 0.3	0.3 ± 0.3
Whole grains, svg/wk	1.2 ± 1.2	1.2 ± 1.2
Red/processed meat, svg/wk	0.9 ± 0.7	0.6 ± 0.5
Fish, svg/wk	0.3 ± 0.3	0.3 ± 0.3
Sugar-sweetened beverage & fruit juice, svg/wk	1.4 ± 1.2	1.1 ± 1.2
Sugar-sweetened beverage, svg/wk	0.5 ± 0.8	0.3 ± 0.6
Fruit juice, svg/wk	0.9 ± 0.9	0.8 ± 0.9
Alcohol, gm/day	15.4 ± 20.0	6.7 ± 11.2
MUFA:SFA	1.1 ± 0.2	1.1 ± 0.2
MUFA, %kcal	11.3 ± 2.7	10.9 ± 2.6
SFA, % kcal	10.5 ± 3.0	10.3 ± 2.8
PUFA, % kcal	5.7 ± 1.7	6.0 ± 1.7
Trans fat, % kcal	1.6 ± 0.8	1.4 ± 0.7
Omega-3 fatty acids, mg/day	257.8 ± 235.5	253.2 ± 217.8

^1^ Values presented as mean and standard deviation or proportion and frequency. Abbreviations: AHEI, Alternative Healthy Eating Index; BMI: body mass index; DASH, Dietary Approaches to Stop Hypertension; MDS, Mediterranean Diet-style Score; MUFA, Monounsaturated fatty acid; PUFA, polyunsaturated fatty acids; SFA, saturated fatty acids.

**Table 2:** Top significant associations of dietary patterns with plasma protein concentrations ^1^.

Name	Type/Class	Diet ^2^	AHEI		DASH		MDS	
			Β ^3^	SE	β	SE	β	SE
Carbonic anhydrase 6	Enzyme	DASH	0.095	0.023	0.108 *	0.023	0.085	0.025
Carnosine dipeptidase 1 (CNDP1)	Peptidase	AHEI, DASH	0.135 *	0.024	0.105 *	0.025	0.085	0.026
Contactin 4	Enzyme	DASH	0.051	0.024	0.112 *	0.024	0.075	0.025
Epidermal growth factor receptor (ERBB1)	Kinase	AHEI, MDS, DASH	0.133 *	0.022	0.127 *	0.023	0.111 *	0.024
Fibroblast growth factor receptor 1 (bFGF-R)	Kinase	AHEI,DASH	0.092 *	0.023	0.107 *	0.023	0.076	0.024
Galectin 3	Other	AHEI	0.101 *	0.023	0.073	0.024	0.076	0.025
Glypican 3	Other	DASH	0.057	0.024	0.109 *	0.024	0.070	0.025
Hemojuvelin BMP co-receptor (RGM-C)	Other	DASH	0.090	0.024	0.105 *	0.025	0.088	0.026
Heterogeneous nuclear ribonucleoprotein A/B (hnRNP A/B)	Enzyme	DASH	−0.054	0.023	−0.099 *	0.024	−0.042	0.025
Kynureninase (KYNU)	Enzyme	AHEI, MDS, DASH	−0.127 *	0.024	−0.153 *	0.024	−0.112 *	0.026
Leucine rich repeats and immunoglobulin like domains 3 (LRIG3)	Other	AHEI	0.114 *	0.024	0.087	0.025	0.069	0.026
Macrophage migration inhibitory factor (MIF)		MDS, DASH	−0.098	0.024	−0.119 *	0.024	−0.106 *	0.026
Matrix metallopeptidase 2 (MMP-2)	Peptidase	DASH	0.076	0.024	0.117 *	0.024	0.074	0.025
MET proto-oncogene, receptor tyrosine kinase (MET)	Kinase	AHEI	0.102 *	0.023	0.096	0.024	0.050	0.025
Notch 1	Transcription regulator	AHEI, DASH	0.098 *	0.023	0.113 *	0.023	0.069	0.024
Peroxiredoxin 1	Enzyme	DASH	−0.068	0.024	−0.113 *	0.025	−0.077	0.026
Plasminogen activator, tissue type (tPA)	Peptidase	DASH	−0.048	0.022	−0.101 *	0.022	−0.068	0.023
Serpin family C member 1 (Antithrombin III)	Enzyme	AHEI	0.093 *	0.021	0.063	0.022	0.053	0.023
Stanniocalcin 1	Kinase	AHEI, MDS, DASH	−0.108 *	0.024	−0.103 *	0.024	−0.111 *	0.025
Thrombospondin 2 (TSP2)	Other	AHEI	−0.112 *	0.024	−0.087	0.025	−0.077	0.026
Transferrin	Transporter	AHEI	0.127 *	0.022	0.083	0.023	0.068	0.024
WAP, follistatin/kazal, immunoglobulin, kunitz and netrin domain containing 2 (WFKN2)	Other	AHEI, DASH	0.095 *	0.023	0.097 *	0.023	0.099	0.024
Prostatic binding protein		DASH	−0.046	0.024	−0.101 *	0.024	−0.042	0.026

^1^ Multivariable regression models with proteins as the dependent variable and dietary pattern scores as the independent variable (separate model for each dietary pattern and protein). Models are adjusted for age, sex, total caloric intake, current smoking, physical activity index, lipid lowering medication, anti-hypertensive medication, and body mass index. * Statistically significant following Bonferroni adjustment (0.05/1373 proteins). ^2^ Diets with a statistically significant association. ^3^ β estimates represent the change in protein concentration per standardized unit increase in the respective dietary pattern indices. Abbreviations: AHEI, Alternative Healthy Eating Index; DASH, Dietary Approaches to Stop Hypertension; MDS, Mediterranean Diet-style Score.

**Table 3:** Top significant associations of dietary patterns with plasma metabolite concentrations ^1.^

Name	Type\Class	Diet ^2^	AHEI		DASH		MDS	
			Β ^3^	SE	β	SE	β	SE
Aconitate	Aliphatic acyclic compound	AHEI, DASH	−0.025 *	0.006	−0.025 *	0.006	−0.019	0.007
Cholesterol ester (C22:6)	Lipid	AHEI, MDS	0.059 *	0.015	0.048	0.016	0.080 *	0.017
Cis/trans-hydroxyproline	L-alpha-amino acid	MDS, DASH	−0.033	0.012	−0.070 *	0.012	−0.054 *	0.013
Cotinine		MDS, DASH	−2.094	0.723	−3.075 *	0.734	−3.097 *	0.772
Hippurate	Aromatic homomonocyclic compounds	AHEI, MDS, DASH	0.364 *	0.079	0.430 *	0.080	0.354 *	0.085
Isocitrate	Aliphatic acyclic compounds	AHEI, MDS, DASH	−0.041 *	0.008	−0.049 *	0.008	−0.041 *	0.008
Lysophosphatidylcholine (C20:5)	Lipid	AHEI, DASH	0.079 *	0.016	0.069 *	0.017	0.062	0.018
Lysophosphatidylcholine (C22:6)	Lipid	AHEI, MDS, DASH	0.073 *	0.010	0.052 *	0.011	0.077 *	0.011
Lysophosphatidylethanolamine (C20:4)	Lipid	MDS	−0.022	0.009	−0.028	0.009	−0.045 *	0.010
Ornithine	L-alpha-amino acid	DASH	−0.028	0.011	−0.057 *	0.011	−0.039	0.012
Oxalate	Aliphatic acyclic compounds	MDS, DASH	0.064	0.018	0.107 *	0.018	0.086 *	0.019
Pantothenate	Water-soluble vitamins	DASH	0.049	0.022	0.126 *	0.022	0.083	0.023
Phosphatidylcholine (C38:6)	Lipid	AHEI, MDS, DASH	0.039 *	0.005	0.035 *	0.005	0.046 *	0.005
Phosphatidylcholine (40:6)	Lipid	AHEI, MDS, DASH	0.041 *	0.006	0.038 *	0.006	0.054 *	0.006
Serine	Amino acid	DASH	−0.006	0.007	−0.030 *	0.007	−0.018	0.008
Sphingomyelin (C14:0)	Lipid	MDS	−0.012	0.006	−0.001	0.007	−0.043 *	0.007
Sphingomyelin (C18:0)	Lipid	AHEI, MDS, DASH	−0.026 *	0.006	−0.028 *	0.006	−0.032 *	0.006
Sphingomyelin (C18:1)	Lipid	AHEI, MDS, DASH	−0.044 *	0.007	−0.044 *	0.007	−0.036 *	0.007
Thiamine	Water-soluble vitamins	DASH	0.067	0.037	0.150 *	0.037	0.101	0.039
Triacylglycerol (C54:7)	Lipid	MDS	0.036	0.012	0.037	0.012	0.050 *	0.013
Triacylglycerol (C56:7)	Lipid	AHEI, MDS, DASH	0.037 *	0.007	0.031 *	0.007	0.041 *	0.008
Triacylglycerol (C56:8)	Lipid	AHEI, MDS, DASH	0.043 *	0.009	0.040 *	0.009	0.054 *	0.009
Triacylglycerol (C58:10)	Lipid	AHEI, MDS, DASH	0.045 *	0.010	0.038 *	0.010	0.048 *	0.011
Triacylglycerol (C58:8)	Lipid	AHEI, MDS, DASH	0.042 *	0.008	0.037 *	0.008	0.047 *	0.008
Triacylglycerol (C58:9)	Lipid	AHEI, MDS, DASH	0.050 *	0.009	0.045 *	0.009	0.057 *	0.009
Uridine	Ribonucleosides	AHEI, DASH	0.028 *	0.005	0.034 *	0.005	0.019	0.005

^1^ Multivariable regression models with each metabolite as the dependent variable and dietary pattern scores as the independent variable (separate model for each dietary pattern and protein). Models are adjusted for age, sex, total caloric intake, current smoking, physical activity index, lipid lowering medication, anti-hypertensive medication, and body mass index. * Statistically significant following Bonferroni adjustment (0.05/216 metabolites). ^2^ Diets with a statistically significant association. ^3^ β estimates represent the change in metabolite concentration per standardized unit increase in the respective dietary pattern indices Abbreviations: AHEI, Alternative Healthy Eating Index; DASH, Dietary Approaches to Stop Hypertension; MDS, Mediterranean Diet-style Score.

**Table 4:** Pathway over representation analysis of protein markers associated with dietary pattern ^1.^

	Pathway	Enrichment Ratio	*P*-Value ^2^	FDR Q-Value ^3^	Matched Molecules
hsa04610	Complement and coagulation cascades	12.51	3.1 × 10^8^	1.0 × 10^5^	PLAT, SERPINC1, C3, SERPINE1, SERPINA5, CFH, C1S, C3AR1, CPB2
hsa05150	Staphylococcus aureus infection	9.81	1.4 × 10^4^	1.7 × 10^2^	C3, CFH, C1S, SELP
hsa01522	Endocrine resistance	6.72	2.5 × 10^4^	1.7 × 10^2^	EGFR, MMP2, BCL2, NOTCH1, MED1, JAG1
hsa04066	HIF-1 signaling pathway	6.59	2.8 × 10^4^	1.7 × 10^2^	EGFR, GAPDH, SERPINE1, TEK, TF, BCL2
hsa05230	Central carbon metabolism in cancer	8.45	2.9 × 10^4^	1.7 × 10^2^	EGFR, FGFR1, KIT, MET, NTRK3
hsa04151	PI3K-Akt signaling pathway	3.41	3.1 × 10^4^	1.7 × 10^2^	CDC37, EGFR, FGFR1, HSP90AB1, MET, TEK, THBS2, BCL2, KIT, YWHAB
hsa05144	Malaria	8.97	9.8 × 10^4^	4.6 × 10^2^	MET, SELE, SELP, THBS2
hsa05418	Fluid shear stress and atherosclerosis	4.74	1.6 × 10^3^	6.2 × 10^2^	HSP90AB1, MMP2, PLAT, BCL2, SELE, BMPR1A
hsa05215	Prostate cancer	5.66	1.8 × 10^3^	6.2 × 10^2^	EGFR, PLAT, HSP90AB1, FGFR1, BCL2
hsa04514	Cell adhesion molecules (CAMs)	4.58	1.9 × 10^3^	6.2 × 10^2^	SELP, SELE, CNTN1, NEGR1, PDCD1LG2, NRXN3

^1^ All proteins analyzed were significantly (FDR q ≤ 0.05) related to the respective dietary pattern scores in multivariable models adjusting for age, sex, total caloric intake, current smoking, physical activity index, lipid lowering medication, anti-hypertensive medication, and body mass index. Analyzed proteins were annotated to KEGG pathways with >5 and <2000 proteins. ^2^ Unadjusted p value. ^3^ False discovery rate p value. Abbreviations: AHEI, Alternative Healthy Eating Index; DASH, Dietary Approaches to Stop Hypertension; KEGG, Kyoto Encyclopedia of Genes and Genomes; MDS, Mediterranean-style Diet Score.

**Table 5:** Overlap among dietary protein quantitative trait loci and prior genome-wide association study risk loci.^1.^

Protein ^1^	Variant ^2^	Nearest Gene	GWAS Associated Traits ^3^	GWAS Study Accession ^4^
AK1A1	rs72688441	NASP	Blood protein concentrations	GCST006585, GCST005806
AK1A1	1:46767127:TCTC_	LRRC41		
AK1A1	rs72684498	ZSWIM5		
AK1A1	rs72676591	EIF2B3		
Antithrombin III	rs334516	TNS3		
bFGF-R	8:38324424:ACC_A	FGFR1		
C1s	rs6695321	CFH	Blood protein concentrations	GCST004365, GCST005806
C1s	rs150845796	CFHR2		
C3a	rs11583804	ACTRT2		
Cadherin-5	rs8176672	ABO	Blood protein concentrations, optic cup area	GCST005806, GCST004137
Cadherin-5	rs11534419	TMEM8C		
Carbonic anhydrase 6	rs11576766	CA6		
Cathepsin D	rs1558500			
Cathepsin S	rs72702561	HORMAD1	Blood protein concentrations	GCST006585
Cathepsin S	rs9661107	PRUNE1		
Cathepsin S	rs141935877	TARS2		
Contactin-1	rs11640313	MRPL28		
ERBB1	rs12112554			
Factor H	rs10737680	CFH	Macular degeneration, lung function, blood protein concentrations	GCST004737, GCST003265, GCST004365
Factor H	rs12134610	KCNT2		
Factor H	rs150845796	CFHR2		
Factor H	rs1329428		Macular degeneration, central serous retinopathy, lung function	GCST000806, GCST006416, GCST003265
Gal 3	rs11733361	SLC25A31		
iC3b	rs11583804	ACTRT2		
IGFBP-1	rs139504202			
KYNU	rs35647509	KYNU		
KYNU	rs4733300	NRG1		
LSAMP	rs117955663	OR56B2P		
MET	rs635634	ABO	Blood protein concentrations, LDL-C concentrations, TC concentrations, ischemic stroke, immune cell count, T2D	GCST000759, GCST000760, GCST006910, GCST004613, GCST004773, GCST005806
Notch 1	rs138015312	UGT2B15		
PARC	rs1102934	CCL18	Blood protein concentrations	GCST006585
RGM-C	rs2381409	FAM221B		
RGM-C	7:57407593:TTCC_			
sE-Selectin	rs2519093	ABO	Blood protein concentrations, LDL-C concentrations, venous thromboembolism, TC concentrations, hematocrit, hemoglobin, immune cell count, CAD	GCST005806, GCST006612, GCST004256, GCST007143, GCST005994, GCST005995, GCST007070, GCST005195
sE-Selectin	rs9722289	TMEM8C		
TIG2	rs10282458	LOC107986858		
Transferrin	rs71544591	PTPRN2		
TSP2	rs73043837	THBS2		
WFKN2	rs7207028	WFIKKN2		

^1^ Proteins whose concentrations were significantly associated (FDR q ≤ 0.05) with at least one dietary pattern index and had a pQTL previously identified in the Framingham Offspring Study [26]. ^2^ Previously identified pQTL. ^3^ Top traits associated with corresponding GWAS risk loci. A complete list of traits can be found in the NHGRI-EBI Catalogue of Published GWAS. ^4^ GWAS Study accession number of studies linking GWAS risk loci and traits. A complete list of GWAS Study accession number can be found in the NHGRI-EBI Catalogue of Published GWAS. CAD, coronary artery disease; GWAS, genome-wide association study; LDL-C, low-density lipoprotein cholesterol; TC, total cholesterol; T2D; diabetes mellitus; QTL, quantitative trait loci.

**Table 6:** Overlap among dietary metabolite quantitative trait loci and prior genome-wide association study risk loci.^1^

Metabolite ^1^	Variant ^2^	Nearest Gene	GWAS Associated Traits ^3^	GWAS Study Accession^4^
Cholesterol ester (C20:5)	rs174548	FADS1-2	Blood metabolite concentrations, metabolite ratios in CKD, TG concentrations, HDL-C concentrations, plasma N6 concentrations, metabolite ratios	GCST000274, GCST005650, GCST000809, GCST000805, GCST003237, GCST002442
Indoxylsulfate	rs875480	CSNK1G3		
Lysophosphatidylcholine (C20:5)	rs174548	FADS1-2	Blood metabolite concentrations, metabolite ratios in CKD, TG concentrations, HDL-C concentrations, plasma N6 concentrations, metabolite ratios	GCST000274, GCST005650, GCST000809, GCST000805, GCST003237, GCST002442
Lysophosphatidylcholine (C22:6)	rs174550	FADS1-2	Blood metabolite concentrations, TG concentrations, FBG, HDL-C concentrations, LDL-C concentrations, plasma N6 concentrations, plasma N3 concentrations, HOMA-B	GCST002443, GCST004237. GCST005186, GCST004232, GCST007141, GCST002446, GCST001178, GCST005180,
Lysophosphatidylethanolamine (C18:2)	rs4246215	FADS2	Plasma N3 concentrations, platelet count, IBD, RBC fatty acid concentrations, Crohn’s disease, CRC	GCST001180, GCST001337, GCST001725, GCST002712, GCST004132, GCST007992
Lysophosphatidylethanolamine (C20:4)	rs174548	FADS1-2	Blood metabolite concentrations, metabolite ratios in CKD, TG concentrations, HDL-C concentrations, plasma N6 concentrations, metabolite ratios	GCST000274, GCST005650, GCST000809, GCST000805, GCST003237, GCST002442
Lysophosphatidylethanolamine (C20:4)	rs4149056	SLCO1B1	Blood metabolite concentrations, TG concentrations, bilirubin, response to statin, ER+ breast cancer, thyroxine concentrations, hemoglobin, metabolite ratios	GCST002442, GCST007133, GCST000386, GCST000213, GCST004359, GCST006896, GCST007068, GCST002442
Phosphatidylcholine (C36:2)	rs174541	FADS2	Blood metabolite concentrations, metabolite ratios in CKD, *trans* fatty acid concentrations, RBC fatty acids,	GCST001852, GCST005650, GCST002721, GCST002712
Phosphatidylcholine (C40:6)	rs174535	FADS1-2	Blood metabolite concentrations, plasma N3 concentrations, RBC fatty acids, *trans* fatty acid concentrations, inflammatory disease, asthma, glycemic traits, respiratory disease	GCST002443, GCST001178, GCST002712, GCST002721, GCST005537, GCST007799, GCST008674, GCST007076
Proline	rs2078743	PRODH		
Serine	rs477992	PHGDH	Blood metabolite/amino acid concentrations, TC concentrations	GCST002966, GCST006614
Sphingomyelin (C14:0)	rs11158519	SYNE2	Sphingomyelin concentrations	GCST008933
Triacylglycerol (C54:2)	rs964184	APOA1/C3/A4/A5	Blood metabolite concentrations, TG concentrations, lipoprotein concentrations, CHD, stroke, MetS, phospholipid fatty acids, fat soluble vitamin concentrations, CAD, age related CVDs	GCST001639, GCST004550, GCST004759, GCST000998, GCST002290, GCST001436, GCST001414, GCST001142, GCST004787, GCST004045,
Triacylglycerol (C54:4)	rs174550	FADS1-2	TG concentrations, FBG, HDL-C concentrations, LDL-C concentrations, plasma N6 concentrations, HOMA-B, plasma N3 concentrations, RBC fatty acids, *trans* fatty acid concentrations	GCST004237, GCST008032, GCST004232, GCST007141, GCST002448, GCST005180, GCST001178, GCST002712, GCST002721
Triacylglycerol (C54:5)	rs964184	APOA1/C3/A4/A5	Blood metabolite concentrations, TG concentrations, lipoprotein concentrations, CHD, stroke,, MetS, phospholipid fatty acids, fat soluble vitamin concentrations, CAD, age related CVDs	GCST001639, GCST004550, GCST004759, GCST000998, GCST002290, GCST001436, GCST001414, GCST001142, GCST004787, GCST004045,
Triacylglycerol (C56:7)	rs6593086			
Triacylglycerol (C58:10)	rs174548	FADS1-2	Blood metabolite concentrations, metabolite ratios in CKD, TG concentrations, HDL-C concentrations, plasma N6 concentrations, metabolite ratios	GCST000274, GCST005650, GCST000809, GCST000805, GCST003237, GCST002442
Triacylglycerol (C58:8)	rs6593086			
Triacylglycerol (C58:9)	rs6593086			

^1^ Metabolites whose concentrations were significantly associated (FDR q ≤ 0.05) with at least one dietary pattern index and had a mQTL previously identified in the Framingham Offspring Study (26). ^2^ Previously identified mQTL. ^3^ Top traits associated with corresponding GWAS risk loci. A complete list of traits can be found in the NHGRI-EBI Catalogue of Published GWAS. ^4^ GWAS Study accession number of studies linking GWAS risk loci and traits. A complete list of GWAS Study accession number can be found in the NHGRI-EBI Catalogue of Published GWAS.

## Supplementary Materials

The following are available online at https://www.mdpi.com/2072-6643/12/5/1476/s1, Supplementary Figure S1. Participant flow diagram, Supplementary Table S1. Statistically significant associations of dietary patterns with plasma protein concentrations, Supplementary Table S2. Statistically significant associations of dietary patterns with plasma metabolite concentrations, Supplementary Table S3. Pathway over representation analysis of protein markers associated with the AHEI and DASH dietary pattern indices, Supplementary Table S4. KEGG pathway mapping of metabolites associated with dietary pattern indices.
