# Supplementary material for: Proteomic and Metabolomic Correlates of Healthy Dietary Patterns: The Framingham Heart Study
Source: Nutrients. 2020 May 19;12(5):1476. doi: 10.3390/nu12051476 (PMC7284467; doi:10.3390/nu12051476)
Supplement: Supplementary file 1 [file nutrients-12-01476-s001.pdf]

Proteomic and Metabolomic Correlates of Healthy Dietary Patterns: The Framingham Heart Study

Walker et al

Online Supplementary Material

Supplementary Figure 1. Participant flow diagram

Supplementary Table 1. Statistically significant associations of dietary patterns with plasma protein concentrations

Supplementary Table 2. Statistically significant associations of dietary patterns with plasma metabolite concentrations

Supplementary Table 3. Pathway over representation analysis of protein markers associated with the AHEI and DASH dietary pattern indices

Supplementary Table 4. KEGG pathway mapping of metabolites associated with dietary pattern indices

Proteomic and Metabolomic Correlates of Healthy Dietary Patterns: The Framingham Heart Study
Walker et al
Online Supplementary Material

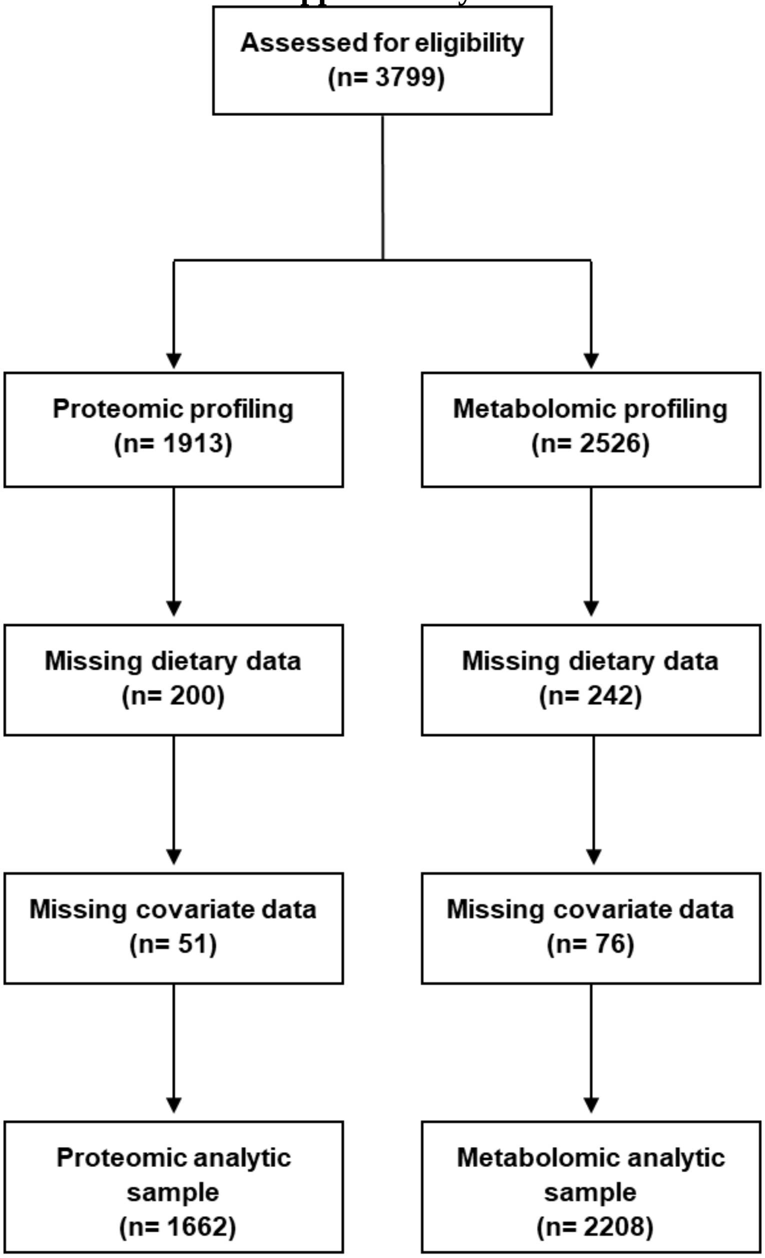

Supplementary Figure 1. Participant flow diagram of the study sample. Among the 3799 participants who attended the fifth examination cycle of the Framingham Offspring Cohort, 1913 participants had blood proteomic profiling completed and 2526 participants had metabolomic profiling completed and were considered eligible for the present investigation. We excluded participants with missing dietary data from each respective analytical sample.

Proteomic and Metabolomic Correlates of Healthy Dietary Patterns: The Framingham Heart Study
Walker et al
Online Supplementary Material

Supplementary Table 1. Statistically significant associations of dietary patterns with plasma protein concentrations[1]

		AHEI			DASH			MDS		
**Label**	**Diet[2]**	**β[3]**	**SE**	**FDR[4]**	**β**	**SE**	**FDR**	**β**	**SE**	**FDR**
14-3-3	AHEI	-0.078	0.024	**0.044**	-0.044	0.025	0.321	-0.046	0.026	0.534
AK1A1	AHEI, DASH	-0.090	0.024	**0.013**	-0.092	0.025	**0.008**	-0.045	0.026	0.570
AMPM2	DASH	-0.060	0.023	0.131	-0.074	0.024	**0.032**	-0.047	0.025	0.490
Antithrombin III	AHEI	0.093	0.021	**0.001**	0.063	0.022	0.053	0.053	0.023	0.379
Apoptosis regulator Bcl-W	AHEI	-0.111	0.033	**0.034**	-0.082	0.033	0.107	-0.063	0.035	0.529
ATS13	DASH	0.065	0.024	0.105	0.089	0.024	**0.009**	0.056	0.025	0.395
bFGF-R	AHEI, DASH	0.092	0.023	**0.004**	0.107	0.023	**0.001**	0.076	0.024	0.125
BMPR1A	DASH	0.065	0.024	0.111	0.086	0.024	**0.013**	0.044	0.025	0.555
C1s	DASH	-0.049	0.022	0.194	-0.073	0.022	**0.024**	-0.047	0.023	0.446
C3a	AHEI	-0.082	0.023	**0.020**	-0.032	0.023	0.488	-0.035	0.025	0.682
Cadherin-5	DASH	0.034	0.024	0.478	0.081	0.025	**0.024**	0.042	0.026	0.605
Calpastatin	DASH	0.045	0.023	0.280	0.069	0.023	**0.050**	0.035	0.024	0.683
Carbonic anhydrase 6	AHEI, DASH	0.095	0.023	**0.004**	0.108	0.023	**0.001**	0.085	0.025	0.069
Cathepsin D	AHEI, DASH	-0.103	0.031	**0.042**	-0.097	0.031	**0.040**	-0.072	0.033	0.410
Cathepsin S	MDS	-0.067	0.025	0.114	-0.073	0.026	0.058	-0.099	0.027	**0.046**
CDC37	DASH	-0.058	0.024	0.178	-0.078	0.025	**0.035**	-0.067	0.026	0.276
CNDP1	AHEI, DASH	0.137	0.024	**0.000**	0.105	0.025	**0.002**	0.085	0.026	0.100
contactin-1	DASH	0.053	0.023	0.178	0.092	0.023	**0.003**	0.084	0.024	0.068
Contactin-4	DASH	0.051	0.024	0.230	0.112	0.024	**0.001**	0.075	0.025	0.158
CRP	DASH	-0.056	0.023	0.167	-0.075	0.023	**0.027**	-0.056	0.024	0.379
Cyclophilin A	AHEI	-0.077	0.024	**0.048**	-0.061	0.025	0.121	-0.055	0.026	0.427

Discoidin domain receptor	AHEI, DASH	0.076	0.024	**0.049**	0.075	0.025	**0.044**	0.056	0.026	0.408
DKK3	DASH	0.062	0.023	0.109	0.090	0.023	**0.005**	0.067	0.024	0.202
dopa decarboxylase	AHEI	0.078	0.024	**0.042**	0.055	0.025	0.174	0.034	0.026	0.728
eIF-5A-1	DASH	-0.063	0.025	0.145	-0.092	0.025	**0.010**	-0.061	0.026	0.379
Elafin	DASH	-0.055	0.024	0.197	-0.083	0.025	**0.020**	-0.085	0.026	0.100
ERBB1	AHEI, DASH, MDS	0.133	0.022	**0.000**	0.127	0.023	**0.000**	0.111	0.024	**0.004**
Esterase D	DASH	-0.058	0.025	0.184	-0.090	0.025	**0.011**	-0.039	0.026	0.659
FABPE	AHEI	-0.078	0.024	**0.042**	-0.062	0.025	0.101	-0.039	0.026	0.649
Factor H	DASH	-0.042	0.021	0.280	-0.066	0.022	**0.042**	-0.043	0.023	0.483
FAM107B	DASH	-0.041	0.023	0.344	-0.080	0.023	**0.018**	-0.031	0.025	0.741
Ferritin	DASH	-0.083	0.030	0.106	-0.121	0.030	**0.003**	-0.059	0.032	0.511
FETUB	DASH	0.065	0.024	0.106	0.084	0.024	**0.014**	0.049	0.025	0.483
Galectin-3	AHEI, DASH	0.101	0.023	**0.001**	0.073	0.024	**0.039**	0.076	0.025	0.142
GAPDH, liver	DASH	-0.047	0.025	0.292	-0.081	0.025	**0.027**	-0.050	0.026	0.488
Glypican 3	DASH	0.057	0.024	0.178	0.109	0.024	**0.001**	0.070	0.025	0.198
hnRNP A/B	DASH	-0.054	0.023	0.184	-0.099	0.024	**0.003**	-0.042	0.025	0.605
hnRNP A2/B1	AHEI, DASH	-0.082	0.024	**0.032**	-0.099	0.025	**0.003**	-0.056	0.026	0.408
HSP 90a/b	AHEI, DASH	-0.092	0.024	**0.013**	-0.090	0.025	**0.011**	-0.056	0.026	0.408
iC3b	AHEI	-0.071	0.023	**0.048**	-0.029	0.023	0.536	-0.052	0.024	0.410
IgD	DASH	-0.050	0.025	0.267	-0.087	0.025	**0.018**	-0.061	0.027	0.379
IGFBP-1	DASH	0.024	0.021	0.593	0.086	0.021	**0.003**	0.042	0.022	0.487
IL-5 Ra	AHEI, DASH	0.079	0.025	**0.047**	0.077	0.026	**0.044**	0.077	0.027	0.167
JAG1	DASH	0.036	0.024	0.436	0.090	0.024	**0.008**	0.055	0.025	0.408

KYNU	AHEI, DASH, MDS	-0.127	0.024	**0.000**	-0.153	0.024	**0.000**	-0.112	0.026	**0.005**
LRIG3	AHEI, DASH	0.114	0.024	**0.001**	0.087	0.025	**0.013**	0.069	0.026	0.238
LSAMP	DASH	0.052	0.020	0.146	0.069	0.021	**0.022**	0.046	0.022	0.412
MDHC	DASH	-0.060	0.024	0.158	-0.099	0.025	**0.003**	-0.055	0.026	0.421
MED-1	AHEI, DASH	0.104	0.032	**0.042**	0.109	0.032	**0.016**	0.075	0.025	0.390
MET	AHEI, DASH	0.102	0.023	**0.001**	0.096	0.024	**0.003**	0.050	0.025	0.449
MFGM	AHEI, DASH	0.086	0.024	**0.021**	0.077	0.025	**0.039**	0.052	0.026	0.473
MIC-1	AHEI, DASH	-0.091	0.028	**0.044**	-0.087	0.028	**0.040**	-0.072	0.030	0.346
MIF	AHEI, DASH, MDS	-0.098	0.024	**0.004**	-0.119	0.024	**0.000**	-0.106	0.026	**0.011**
MMP-2	AHEI, DASH	0.076	0.024	**0.044**	0.117	0.024	**0.000**	0.074	0.025	0.167
Myokinase, human	DASH	-0.050	0.025	0.262	-0.099	0.025	**0.004**	-0.049	0.026	0.498
NCAM-120	DASH	0.049	0.022	0.206	0.070	0.022	**0.035**	0.068	0.023	0.158
NEGR1	DASH	0.066	0.031	0.231	0.094	0.031	**0.044**	0.075	0.033	0.386
Notch 1	AHEI, DASH	0.098	0.023	**0.001**	0.113	0.023	**0.000**	0.069	0.024	0.167
NRX3B	AHEI	0.089	0.024	**0.013**	0.071	0.024	0.053	0.062	0.026	0.319
PAI-1	DASH	-0.043	0.022	0.282	-0.067	0.023	**0.050**	-0.014	0.024	0.906
PARC	DASH	-0.071	0.023	0.061	-0.080	0.024	**0.019**	-0.068	0.025	0.208
PCI	AHEI	0.086	0.023	**0.015**	0.045	0.024	0.288	0.040	0.025	0.609
PD-L2	AHEI, DASH	0.083	0.024	**0.032**	0.074	0.025	**0.049**	0.041	0.026	0.609

Peroxiredoxin-1	DASH	-0.068	0.024	0.099	-0.113	0.025	**0.001**	-0.077	0.026	0.158
Peroxiredoxin-6	AHEI	-0.078	0.024	**0.044**	-0.067	0.025	0.071	-0.052	0.026	0.449
PGCB	AHEI, DASH	0.071	0.022	**0.044**	0.075	0.023	**0.021**	0.053	0.024	0.386
PGM1	AHEI	-0.116	0.033	**0.021**	-0.025	0.033	0.761	-0.050	0.035	0.683
P-Selectin	MDS	-0.044	0.024	0.317	-0.060	0.025	0.124	-0.094	0.026	**0.048**
PLPP	AHEI, DASH	-0.079	0.025	**0.047**	-0.094	0.025	**0.008**	-0.065	0.027	0.323
PolyUbiquitin K48	DASH	-0.034	0.031	0.604	-0.104	0.030	**0.018**	-0.073	0.033	0.386
prostatic binding protein	DASH	-0.046	0.024	0.288	-0.101	0.024	**0.003**	-0.042	0.026	0.605
PSA1	AHEI	-0.074	0.023	**0.044**	-0.063	0.024	0.081	-0.031	0.025	0.744
Rab GDP dissociation inhi	DASH	-0.072	0.024	0.065	-0.089	0.025	**0.011**	-0.054	0.026	0.429
RBP	DASH	0.087	0.032	0.112	0.095	0.032	**0.050**	0.084	0.034	0.318
RGMA	AHEI, DASH	0.103	0.031	**0.042**	0.098	0.031	**0.035**	0.067	0.033	0.449
RGMB	DASH	0.059	0.024	0.156	0.073	0.024	**0.044**	0.049	0.025	0.483
RGM-C	AHEI, DASH	0.090	0.024	**0.013**	0.105	0.025	**0.002**	0.088	0.026	0.070
SCF sR	AHEI	0.079	0.022	**0.021**	0.058	0.023	0.106	0.047	0.024	0.482
sE-Selectin	DASH	-0.063	0.024	0.117	-0.089	0.024	**0.009**	-0.082	0.025	0.100
sICAM-5	MDS	-0.040	0.023	0.364	-0.057	0.024	0.125	-0.089	0.025	**0.048**
SLAF6	DASH	-0.072	0.023	0.055	-0.076	0.024	**0.028**	-0.080	0.025	0.107
SNAA	DASH	-0.058	0.025	0.184	-0.081	0.025	**0.028**	-0.035	0.026	0.726
Stanniocalcin-1	AHEI, DASH, MDS	-0.108	0.024	**0.001**	-0.103	0.024	**0.002**	-0.111	0.025	**0.005**
STAT6	AHEI	-0.107	0.033	**0.044**	-0.052	0.034	0.407	-0.064	0.036	0.526
sTie-1	DASH	0.075	0.024	0.055	0.099	0.025	**0.003**	0.066	0.026	0.291
sTie-2	DASH	0.062	0.025	0.150	0.081	0.025	**0.027**	0.066	0.026	0.311
TAFI	AHEI	0.083	0.024	**0.028**	0.037	0.025	0.420	0.060	0.026	0.361
TECK	DASH	-0.020	0.025	0.713	-0.099	0.025	**0.004**	-0.070	0.026	0.250

TIG2	DASH	-0.068	0.022	0.055	-0.090	0.022	**0.003**	-0.081	0.023	0.069
tPA	DASH	-0.048	0.022	0.206	-0.101	0.022	**0.001**	-0.068	0.023	0.158
Transferrin	AHEI, DASH	0.127	0.022	**0.000**	0.083	0.023	**0.011**	0.068	0.024	0.181
Transketolase	DASH	-0.070	0.025	0.089	-0.084	0.025	**0.020**	-0.043	0.026	0.605
TrATPase	DASH	-0.048	0.021	0.203	-0.067	0.022	**0.040**	-0.036	0.023	0.609
Triosephosphate isomerase	DASH	-0.065	0.025	0.117	-0.083	0.025	**0.024**	-0.042	0.026	0.609
TrkB	DASH	0.068	0.024	0.101	0.090	0.025	**0.010**	0.061	0.026	0.370
TrkC	AHEI, DASH	0.073	0.023	**0.048**	0.076	0.024	**0.029**	0.061	0.025	0.318
TSP2	AHEI, DASH	-0.112	0.024	**0.001**	-0.087	0.025	**0.014**	-0.077	0.026	0.158
TWEAK	AHEI	0.086	0.024	**0.016**	0.070	0.024	0.053	0.057	0.025	0.386
TYK2	DASH	-0.043	0.024	0.317	-0.086	0.024	**0.011**	-0.062	0.025	0.318
UBC9	AHEI, DASH	-0.079	0.025	**0.044**	-0.088	0.025	**0.014**	-0.067	0.026	0.304
UBE2N	DASH	-0.075	0.025	0.064	-0.099	0.025	**0.004**	-0.049	0.027	0.512
Ubiquitin+1	DASH	-0.059	0.024	0.170	-0.101	0.025	**0.003**	-0.051	0.026	0.473
WFKN2	AHEI, DASH, MDS	0.095	0.023	**0.003**	0.097	0.023	**0.002**	0.099	0.024	**0.011**

[1]Multivariable regression models with proteins as the dependent variable and dietary pattern scores as the independent variable (separate model for each dietary pattern and protein). Models are adjusted for age, sex, total caloric intake, current smoking, physical activity index, lipid lowering medication, anti-hypertensive medication, and body mass index.
[2]Diets with a statistically significant association.
[3]β estimates represent the change in protein concentrations per standardized unit increase in the respective dietary pattern indices.
[4]False discovery rate p value. Statistically significant (FDR q ≤0.05) values are in bold.
Abbreviations: AHEI, Alternative Healthy Eating Index; DASH, Dietary Approaches to Stop Hypertension; MDS, Mediterranean Diet-style Score.

Proteomic and Metabolomic Correlates of Healthy Dietary Patterns: The Framingham Heart Study

Walker et al

Online Supplementary Material

Supplementary Table 2. Statistically significant associations of dietary patterns with plasma metabolite concentrations[1]

		AHEI			DASH			MDS		
**Label**	**Diet[2]**	**β[3]**	**SE**	**FDR[4]**	**β**	**SE**	**FDR**	**β**	**SE**	**FDR**
3-OH-anthranilic acid	DASH	0.022	0.010	0.130	0.032	0.011	**0.015**	0.017	0.011	0.295
Aconitate	AHEI, DASH, MDS	-0.025	0.006	**0.001**	-0.025	0.006	**0.001**	-0.019	0.007	**0.021**
Adenosine diphosphate (ADP)	AHEI, MDS	-0.092	0.034	**0.038**	-0.064	0.034	0.170	-0.068	0.036	**0.181**
Adenosine monophosphate (AMP)	MDS	-0.356	0.156	0.088	-0.340	0.158	0.111	-0.496	0.168	**0.018**
betaine	DASH, MDS	0.004	0.004	0.529	0.012	0.004	**0.014**	0.014	0.004	**0.005**
Cholesterol ester (C20:5)	AHEI, DASH, MDS	0.049	0.015	**0.015**	0.044	0.016	**0.024**	0.054	0.017	**0.009**
Cholesterol ester (C22:6)	AHEI, DASH, MDS	0.059	0.015	**0.002**	0.048	0.016	**0.014**	0.080	0.017	**0.000**
choline	AHEI, DASH	-0.012	0.004	**0.045**	-0.014	0.005	**0.012**	-0.006	0.005	0.353
cis/trans-hydroxyproline	AHEI, DASH, MDS	-0.033	0.012	**0.039**	-0.070	0.012	**0.000**	-0.054	0.013	**0.000**
cotinine	AHEI, DASH, MDS	-2.094	0.723	**0.031**	-3.075	0.734	**0.000**	-3.097	0.772	**0.001**
Deoxycholates	AHEI, DASH, MDS	-0.088	0.031	**0.031**	-0.092	0.031	**0.018**	-0.055	0.033	0.237
Fructose-1-phosphate + fructose-6-phosphate + glucose-1-phosphate + glucose-6-phosphate	AHEI	-0.033	0.011	**0.031**	-0.001	0.012	0.976	-0.004	0.012	0.856
Glyco deoxycholates	DASH	-0.089	0.034	0.050	-0.113	0.034	**0.009**	-0.086	0.037	0.073

Glycocholate	AHEI, DASH, MDS	-0.080	0.029	**0.038**	-0.095	0.029	**0.011**	-0.084	0.031	**0.033**
Hippurate	AHEI, DASH, MDS	0.364	0.079	**0.000**	0.430	0.080	**0.000**	0.354	0.085	**0.000**
Indoxylsulfate	DASH	-0.036	0.015	0.075	-0.048	0.015	**0.011**	-0.034	0.016	0.112
Inositol	DASH, MDS	0.008	0.008	0.547	0.026	0.008	**0.015**	0.029	0.009	**0.010**
Isocitrate	AHEI, DASH, MDS	-0.041	0.008	**0.000**	-0.049	0.008	**0.000**	-0.041	0.008	**0.000**
lysine	AHEI	0.019	0.007	**0.033**	0.015	0.007	0.118	0.016	0.007	0.109
Lysophosphatidylcholine (C18:1)	AHEI, MDS	-0.031	0.011	**0.031**	-0.024	0.011	0.106	-0.029	0.011	**0.044**
Lysophosphatidylcholine (C20:5)	AHEI, DASH, MDS	0.079	0.016	**0.000**	0.069	0.017	**0.000**	0.062	0.018	**0.004**
Lysophosphatidylcholine (C22:6)	AHEI, DASH, MDS	0.073	0.010	**0.000**	0.052	0.011	**0.000**	0.077	0.011	**0.000**
Lysophosphatidylethanolamine (C18:1)	MDS	-0.030	0.015	0.138	-0.035	0.015	0.082	-0.045	0.016	**0.023**
Lysophosphatidylethanolamine (C18:2)	MDS	-0.023	0.010	0.088	-0.025	0.010	0.070	-0.034	0.011	**0.011**
Lysophosphatidylethanolamine (C20:4)	DASH, MDS	-0.022	0.009	0.075	-0.028	0.009	**0.016**	-0.045	0.010	**0.000**
Malate	AHEI, DASH, MDS	-0.021	0.007	**0.031**	-0.023	0.007	**0.012**	-0.025	0.008	**0.008**
ornithine	DASH, MDS	-0.028	0.011	0.059	-0.057	0.011	**0.000**	-0.039	0.012	**0.008**
Oxalate	AHEI, DASH, MDS	0.064	0.018	**0.004**	0.107	0.018	**0.000**	0.086	0.019	**0.000**

Pantothenate	DASH, MDS	0.049	0.022	0.088	0.126	0.022	**0.000**	0.083	0.023	**0.004**
Phosphatidylcholine (C32:1)	MDS	-0.020	0.012	0.231	-0.003	0.012	0.892	-0.042	0.013	**0.008**
Phosphatidylcholine (C34:1)	MDS	-0.005	0.004	0.308	-0.004	0.004	0.503	-0.012	0.004	**0.016**
Phosphatidylcholine (C36:1)	MDS	-0.015	0.006	0.062	-0.006	0.006	0.565	-0.018	0.007	**0.038**
Phosphatidylcholine (C36:2)	AHEI, DASH	-0.010	0.003	**0.008**	-0.009	0.003	**0.016**	-0.007	0.003	0.086
Phosphatidylcholine (C38:6)	AHEI, DASH, MDS	0.039	0.005	**0.000**	0.035	0.005	**0.000**	0.046	0.005	**0.000**
Phosphatidylcholine (C40:6)	AHEI, DASH, MDS	0.041	0.006	**0.000**	0.038	0.006	**0.000**	0.054	0.006	**0.000**
Phosphoglycerate	AHEI	-0.044	0.016	**0.038**	-0.035	0.016	0.118	-0.022	0.017	0.405
proline	DASH	-0.007	0.006	0.376	-0.015	0.006	**0.037**	-0.011	0.006	0.197
serine	DASH	-0.006	0.007	0.577	-0.030	0.007	**0.000**	-0.018	0.008	0.062
Sphingomyelin (C14:0)	MDS	-0.012	0.006	0.173	-0.010	0.007	0.316	-0.043	0.007	**0.000**
Sphingomyelin (C16:0)	DASH, MDS	-0.006	0.003	0.088	-0.009	0.003	**0.012**	-0.008	0.003	**0.021**
Sphingomyelin (C18:0)	AHEI, DASH, MDS	-0.026	0.006	**0.000**	-0.028	0.006	**0.000**	-0.032	0.006	**0.000**
Sphingomyelin (C18:1)	AHIE, DASH, MDS	-0.044	0.007	**0.000**	-0.044	0.007	**0.000**	-0.036	0.007	**0.000**
thiamine	DASH, MDS	0.067	0.037	0.187	0.150	0.037	**0.001**	0.101	0.039	**0.046**
threonine	DASH	-0.005	0.006	0.550	-0.015	0.006	**0.043**	-0.010	0.006	0.252
Triacylglycerol (C48:1)	MDS	-0.027	0.015	0.175	-0.015	0.015	0.510	-0.043	0.016	**0.027**
Triacylglycerol (C50:1)	MDS	-0.019	0.009	0.131	-0.011	0.009	0.461	-0.028	0.010	**0.025**
Triacylglycerol (C50:2)	AHEI, MDS	-0.021	0.007	**0.031**	-0.015	0.007	0.118	-0.026	0.008	**0.007**
Triacylglycerol (C52:2)	AHEI, DASH	-0.020	0.006	**0.013**	-0.017	0.006	**0.038**	-0.014	0.007	0.112

Triacylglycerol (C54:1)	DASH, MDS	-0.101	0.040	0.060	-0.108	0.040	**0.037**	-0.118	0.043	**0.030**
Triacylglycerol (C54:2)	AHEI, DASH, MDS	-0.056	0.019	**0.030**	-0.055	0.019	**0.022**	-0.056	0.020	**0.030**
Triacylglycerol (C54:4)	MDS	-0.003	0.007	0.773	0.001	0.007	0.934	0.019	0.007	**0.040**
Triacylglycerol (C54:5)	MDS	0.009	0.008	0.416	0.011	0.008	0.350	0.029	0.008	**0.005**
Triacylglycerol (C54:6)	MDS	0.009	0.008	0.416	0.011	0.008	0.350	0.029	0.008	**0.005**
Triacylglycerol (C54:7)	AHEI, DASH, MDS	0.036	0.012	**0.019**	0.037	0.012	**0.014**	0.050	0.013	**0.001**
Triacylglycerol (C56:6)	AHEI, DASH, MDS	0.017	0.006	**0.034**	0.012	0.006	0.144	0.018	0.007	**0.029**
Triacylglycerol (C56:7)	AHEI, DASH, MDS	0.037	0.007	**0.000**	0.031	0.007	**0.000**	0.041	0.008	**0.000**
Triacylglycerol (C56:8)	AHEI, DASH, MDS	0.043	0.009	**0.000**	0.040	0.009	**0.000**	0.054	0.009	**0.000**
Triacylglycerol (C56:9)	AHEI, MDS	0.033	0.010	**0.009**	0.022	0.010	0.111	0.034	0.011	**0.009**
Triacylglycerol (C58:10)	AHEI, DASH, MDS	0.045	0.010	**0.000**	0.038	0.010	**0.001**	0.048	0.011	**0.000**
Triacylglycerol (C58:8)	AHEI, DASH, MDS	0.042	0.008	**0.000**	0.037	0.008	**0.000**	0.047	0.008	**0.000**
Triacylglycerol (C58:9)	AHEI, DASH, MDS	0.050	0.009	**0.000**	0.045	0.009	**0.000**	0.057	0.009	**0.000**
Triacylglycerol (C60:12)	AHEI, MDS	0.043	0.014	**0.014**	0.030	0.014	0.106	0.045	0.015	**0.013**
Tryptophan	DASH, MDS	0.009	0.004	0.149	0.014	0.004	**0.014**	0.015	0.005	**0.011**

Uridine	AHEI, DASH, MDS	0.028	0.005	**0.000**	0.034	0.005	**0.000**	0.019	0.005	**0.004**
Uridine diphosphate-galactose + uridine diphosphate-glucose	AHEI	-0.046	0.017	**0.037**	-0.033	0.017	0.150	-0.045	0.018	0.055

[1]Multivariable regression models with each metabolite as the dependent variable and dietary pattern scores as the independent variable (separate model for each dietary pattern and protein). Models are adjusted for age, sex, total caloric intake, current smoking, physical activity index, lipid lowering medication, anti-hypertensive medication, and body mass index.
[2]Diets with a statistically significant association.
[3]β estimates represent the change in metabolite concentration per standardized unit increase in the respective dietary pattern indices.
[4]False discovery rate p value. Statistically significant (FDR q ≤0.05) values are in bold.
Abbreviations: AHEI, Alternative Healthy Eating Index; DASH, Dietary Approaches to Stop Hypertension; MDS, Mediterranean Diet-style Score.

.

Supplementary Table 3. **Pathway over representation analysis of protein markers associated with the AHEI and DASH dietary pattern indices[1]**

AHEI	Pathway	Enrichment Ratio	p value[2]	FDR q value[3]	Matched Molecules
	Central carbon metabolism in cancer	15.12	1.7E-05	5.6E-03	EGFR, FGFR1, KIT, MET, NTRK3
	Complement and coagulation cascades	9.95	6.5E-04	8.0E-02	SERPINC1, C3, C3AR1, CPB2
	MicroRNAs in cancer	6.55	8.9E-04	8.0E-02	EGFR, MET, NOTCH1, BCL2L2, UBE2I
	Pathways in cancer	3.38	9.8E-04	8.0E-02	EGFR, FGFR1, HSP90AB1, IL5RA, KIT, MET, MMP2, NOTCH1, STAT6
	Endocrine resistance	8.02	1.5E-03	9.5E-02	EGFR, MMP2, NOTCH1, MED1
	Phenylalanine metabolism	23.12	3.3E-03	1.8E-01	MIF, DDC
	Estrogen signaling pathway	5.74	4.9E-03	2.1E-01	EGFR, CTSD, HSP90AB1, MMP2
	Adherens junction	8.19	5.7E-03	2.1E-01	EGFR, MET, FGFR1
	Melanoma	8.19	5.7E-03	2.1E-01	EGFR, MET, FGFR1
	Breast cancer	5.35	6.3E-03	2.1E-01	EGFR, KIT FGFR1, NOTCH1
**DASH**					
	Endocrine resistance	7.57	4.8E-04	8.4E-02	JAG1, EGFR, MMP2, NOTCH1, MED1
	HIF-1 signaling pathway	7.42	5.3E-04	8.4E-02	EGFR, GAPDH, SERPINE1, TEK, TF
	Central carbon metabolism in cancer	9.13	9.1E-04	8.4E-02	EGFR, FGFR1, MET, NTRK3
	Carbon metabolism	6.39	1.0E-03	8.4E-02	ESD, GAPDH, MDH1, TKT, TPI1
	Complement and coagulation cascades	7.51	1.9E-03	1.1E-01	PLAT, SERPINC1, CFH, C1S
	Fluid shear stress and atherosclerosis	5.38	2.2E-03	1.1E-01	HSP90AB1, MMP2, PLAT, SELE, BMPR1A
	PI3K-Akt signaling pathway	3.35	2.3E-03	1.1E-01	CDC37, EGFR, FGFR1, HSP90AB1, MET, NTRK2, TEK, THBS2
	Prostate cancer	6.12	4.0E-03	1.5E-01	EGFR, PLAT, HSP90AB1, FGFR1
	Malaria	9.09	4.3E-03	1.5E-01	MET, SELE, THBS2

Proteoglycans in cancer	3.75	1.0E-02	3.0E-01	EGFR, FGFR1, GPC3, MET, MMP2

[1]All proteins analyzed were significantly (FDR $p \leq 0.05$) related to the respective dietary pattern scores in multivariable models adjusting for age, sex, total caloric intake, current smoking, physical activity index, lipid lowering medication, anti-hypertensive medication, and body mass index. Analyzed proteins were annotated to KEGG pathways with > 5 and < 2000 proteins.

[2]Unadjusted p value

[3]False discovery rate q value.

Abbreviations: AHEI, Alternative Healthy Eating Index; DASH, Dietary Approaches to Stop Hypertension; KEGG, Kyoto Encyclopedia of Genes and Genomes; MDS, Mediterranean-style Diet Score

Proteomic and Metabolomic Correlates of Healthy Dietary Patterns: The Framingham Heart Study
Walker et al
Online Supplementary Material

Supplementary Table 4. KEGG pathway mapping of metabolites associated with dietary pattern indices[1]

AHEI	Pathway	Total Molecules	Matched Molecules
	Metabolic pathways	11	ADP, Choline, Phosphatidylcholine, Uridine, Isocitrate, cis-Aconitate , Triacylglycerol, Sphingomyelin, D-Lysine, Hiipurate, Glycocholate
	Bile secretion	4	Choline, Glycocholate, Cholesterol ester, Deoxycholic acid
	Glycerophospholipid metabolism	3	Choline, Phosphatidylcholine, 1-Acyl-sn-glycero-3-phosphochiline
	Cholesterol metabolism	3	Choline, Phosphatidylcholine, 1-Acyl-sn-glycero-3-phosphochiline
	Choline metabolism in cancer	3	Triacylglycerol, Glycocholate, Cholesterol ester
**DASH**			
	Metabolic pathways	16	L-Serine, L-Ornithine, Choline, Phosphatidylcholine, L-Threonine, Uridine, Isocitrate, Thiamine, cis-Aconitate, Sphingomyelin, 3-Hydroxyanthranilate, Betaine, D-Proline, Pantothenate, Hippurate, Glycocholate
	ABC transporters	7	L-Serine, L-Ornithine, Choline, L-Threonine, Uridine, Thiamine, Betaine
	Biosynthesis of amino acids	4	L-Serine, L-Ornithine, L-Threonine, Isocitrate
	Bile secretion	4	Choline, Glycocholate, Cholesterol ester, Deoxycholic acid
	Glycine, serine and threonine metabolism	4	L-Serine, L-Ornithine, L-Threonine, Betaine
	Glyoxylate and dicarboxylate metabolism	3	L-Serine, Isocitrate, cis-Aconitate
	Vitamin digestion and absorption	3	Thiamine, Pantothenate, Cholesterol ester
	Choline metabolism in cancer	3	Choline, Phosphatidylcholine, 1-Acyl-sn-glycero-3-phosphochiline
	2-Oxocarboxylic acid metabolism	3	L-Ornithine, Isocitrate, cis-Aconitate,
	Glycerophospholipid metabolism	3	Choline, Phosphatidylcholine, 1-Acyl-sn-glycero-3-phosphochiline

**MDS**			
	Metabolic pathways	13	AMP, L-Ornithine, Phosphatidylcholine, Uridine, Isocitrate, Thiamine, cis-Aconitate, Triacylglycerol, Sphingomyelin, Betaine, Pantothenate, Hippurate, Glycocholate
	Vitamin digestion and absorption	4	Thiamine, Triacylglycerol, Pantothenate, Cholesterol ester
	Oxocarboxylic acid metabolism	3	L-Ornithine, Isocitrate, cis-Aconitate
	ABC transporters	4	L-Ornithine, Uridine, Thiamine, Betaine
	Cholesterol metabolism	3	Triacylglycerol, Glycocholate, Cholesterol ester

[1]Pathways with ≥3 mapped metabolites are displayed. All metabolites assessed were significantly (FDR $p < 0.05$) associated with the respective dietary pattern scores in multivariable models adjusting for age, sex, total caloric intake, current smoking, physical activity index, lipid lowering medication, anti-hypertensive medication, and body mass index. Abbreviations: AHEI, Alternative Healthy Eating Index; DASH, Dietary Approaches to Stop Hypertension; FDR, false discovery rate; KEGG, Kyoto Encyclopedia of Genes and Genomes; MDS, Mediterranean Diet Score.
